# Ethical Considerations for Gene Drive: Challenges of Balancing Inclusion, Power and Perspectives

**DOI:** 10.3389/fbioe.2022.826727

**Published:** 2022-01-21

**Authors:** Ana Kormos, Gregory C. Lanzaro, Ethan Bier, Vanilson Santos, Lodney Nazaré, João Pinto, Adionilde Aguiar dos Santos, Anthony A. James

**Affiliations:** ^1^ Vector Genetics Laboratory, University of California, Davis, Davis, CA, United States; ^2^ Section of Cell and Developmental Biology, University of California, San Diego, San Diego, CA, United States; ^3^ Ministry of Defense, São Tomé, São Tomé and Príncipe; ^4^ United Nations Development Program, São Tomé, São Tomé and Príncipe; ^5^ Global Health and Tropical Medicine, Instituto de Higiene e Medicina Tropical, Universidade Nova de Lisboa, Lisbon, Portugal; ^6^ Ministry of Health, Delegacia de Saúde Distrital de Água Grande, São Tomé, São Tomé and Príncipe; ^7^ Department of Microbiology and Molecular Genetics, University of California, Irvine, CA, Irvine, United States; ^8^ Department of Molecular Biology and Biochemistry, University of California, Irvine, CA, Irvine, United States

**Keywords:** gene drive, ethics, engagement, co-development, responsible research, research guidelines

## Abstract

Progress in gene-drive research has stimulated discussion and debate on ethical issues including community engagement and consent, policy and governance, and decision-making involved in development and deployment. Many organizations, academic institutions, foundations, and individual professionals have contributed to ensuring that these issues are considered prior to the application of gene-drive technology. Central topics include co-development of the technology with local stakeholders and communities and reducing asymmetry between developers and end-users. Important questions include with whom to conduct engagement and how to define community acceptance, develop capacity-building activities, and regulate this technology. Experts, academics, and funders have suggested that global frameworks, standards, and guidelines be developed to direct research in answering these important questions. Additionally, it has been suggested that ethical principles or commitments be established to further guide research practices. The challenging and interesting contradiction that we explore here is that the vast majority of these conversations transpire with little or no input from potential end-users or stakeholders who, we contend, should ultimately determine the fate of the technology in their communities. The question arises, whose concerns regarding marginalization, disempowerment, and inequity should be included in discussions and decisions concerning how inequities are perceived and how they may be addressed? At what stage will true co-development occur and how will opinions, perspectives and knowledge held by low-income country stakeholders be applied in determining answers to the questions regarding the ethics being debated on the academic stage? Our opinion is that the time is now.

## Introduction

The University of California Irvine Malaria Initiative (UCIMI), a not-for-profit research collaborative, has been actively involved in many of the discussions, workshops, and seminars addressing the application of gene-drive technology. The UCIMI mission is to contribute to malaria eradication through population modification of the African malaria vector mosquitoes, *Anopheles gambiae,* and *An. coluzzii,* rendering them incapable of transmitting malaria parasites to humans (Carballar-Lejarazú et al., 2020). Our perspectives here represent those of a research program that has developed gene-drive systems for public health application using a relationship-based approach (Kormos et al., 2020).

A simple internet search for “gene drive” will demonstrate the large number of perspectives surrounding this technology and the questions of whether and when to apply it. Numerous publications explore these questions, and many also offer conceptual frameworks, guidance, or ethical considerations for developers of gene drive (Lavery et al., 2010; Ramsey et al., 2014; WHO Special Programme for Research and Training in Tropical Diseases, 2014; National Academies of Sciences Engineering and Medicine, 2016; Emerson et al., 2017; Roberts et al., 2017; AUDA-NEPAD, 2018; Collins, 2018; James et al., 2018; Brossard et al., 2019; Thizy et al., 2019; Deplazes-Zemp et al., 2020; World Health Organization, 2020; Annas et al., 2021; World Health Organization, 2021). These guidelines are developed for broad application, to be adapted to local environments. This alone poses challenges associated with political, social, regulatory and environment complexities and differences from one place to another. We believe that guidance should be developed on a case-by-case basis for application in alignment with the relationship-based model (Kormos et al., 2020). However, we do not focus here on this point, rather we argue that in the development of guidance, whether developed locally or globally, it is critically important to consider whose perspectives and values are included in the development process.

In addition to publications, there are institutions and organizations exploring these questions through workshops and webinars; gathering experts and academics together to discuss perspectives and ideas on the topic. For example, in 2021 the Gene Convene Global Collaborative hosted a series of virtual panel discussions entitled “Considering the case of Gene Drive Technologies Through Social Science Theories on Stakeholder Engagement” and a second session under the heading, “Unsettled Ethical Issues in Gene Drive Research.”

With few exceptions, the primary authors, organizations, and institutions engaged in these discussions, like gene-drive developers themselves, are not located in areas where the technology is being proposed for use to address public health concerns. The voices and values represented are largely those of individuals who are not living in communities that will be directly impacted by the application of this technology. Additionally, many of these academic experts are not working directly with practitioners in the field. This is the interesting challenge we would like to explore further here; the increasing divergence between academic and theoretical recommendations and those of the practitioners engaged directly with stakeholders and community members. This difference in understanding presents several specific areas of concern: 1) Perspectives - the voices that are front-and-center in these discussions do not necessarily represent the perspectives and values of those who share the greatest risk/benefit of the application of the technology, 2) Inclusion - while emphasizing the importance of co-development, much of the proposed guidance and recommendations is not co-developed with stakeholders, and 3) Power—the current system of decision-making and knowledge production enhances the imbalance of power and restricts illumination of important local perspectives and insights.

The uncertainty of outcomes associated with the application/testing of this novel technology has created an expansive niche for academic exploration of the important ethical and moral questions associated with it. While these are critically important issues to consider, it is just as important, and we would argue necessary, to evaluate whose values and voices ought to be driving the answers to these questions.

## Perspectives: Voices and Values Influencing Gene Drive Research

Proposed guidelines, principles, and commitments for gene-drive research present recommendations for ethically-responsible development and deployment practices for researchers (Lavery et al., 2010; Ramsey et al., 2014; WHO Special Programme for Research and Training in Tropical Diseases, 2014; National Academies of Sciences Engineering and Medicine, 2016; Emerson et al., 2017; Roberts et al., 2017; AUDA-NEPAD, 2018; Collins, 2018; James et al., 2018; Brossard et al., 2019; Thizy et al., 2019; Deplazes-Zemp et al., 2020; Long et al., 2020; World Health Organization, 2020; Annas et al., 2021; World Health Organization, 2021). It is critically important that application of the technology follow practices of social responsibility, transparency, accountability, and compliance with local governance and regulatory infrastructure. If we are to create guidance frameworks intended for widespread application, it also is important to consider whose perspectives helped develop the principles and guidelines meant to ensure these practices.

A quick review of the authors and associated institutions and organizations of most published recommendations reveal that this work is led largely by academics and experts who are not engaged directly in research and engagement activities at proposed field sites.

The same situation pertains to workshops and symposia designed to bring diverse perspectives together to discuss important questions and challenges surrounding gene-drive research, such as whom, when and how to conduct engagement, how to define and determine community consent/acceptance, and what ethical principles should guide responsible research. These broad and complex topics are typically led and facilitated by academic and professional experts who offer important insights in these areas. While discussions lead to an expanded list of questions and considerations for gene-drive research, too often, voices and values represented in these venues are not representative of those who are regularly engaged with communities and stakeholders in the field, or directly involved in managing and conducting research at field sites. In addition to providing important insights about diverse, often complex, community perspectives and values, stakeholders and community leaders also offer understanding about local governance and regulation that will likely impact and influence how such guidance is implemented. Individuals who are closest to the work in the field and grappling actively with these challenges are under-represented. Ethically-responsible development of this technology is especially important for public health applications where guarantee of the communities’ best interests, and respect for their ideology and values, need to be factored into the principles guiding the work. Collaborations with existing ethics committees, social scientists, public health professionals, and biosafety/biotechnology regulators from field sites offer essential perspectives to help guide best practices and responsible development of the technology. It has been suggested previously that regulators from field sites should be involved directly in the facilitation and adaptation of essential guidelines and frameworks for the technology (AUDA-NEPAD, 2018).

If we are to create guidance and recommendations for widespread implementation and to influence research practice and ensure ethically-responsible development, is it not essential to involve the perspectives of those most directly involved? Involvement requires intentional planning and communication with developers and their collaborators at field-sites to build a connection with appropriate stakeholders. It necessitates rigorous reciprocal engagement between developers and stakeholders for exchange of knowledge and technical capacity building to support advanced understanding of genetic principles and molecular biology that apply to the technology. It requires stakeholder invitation to participate in meetings, workshops, and in the collaborative writing process. It calls for collaboration with groups who may not have the same experience and knowledge but whose opinions need to be taken into account so that they are part of the solution. Without the collaboration of field-site stakeholders and field practitioners, we risk creating recommendations lacking representation of the voices and values of entire groups for whom the recommendations are designed in large part to protect and support.

## Inclusion: Activating the Concept of Co-Development

Co-develop is defined as “to develop (something) by working with one or more others to develop (something) jointly” (Merriam-Webster, 2021). Co-development is articulated as an important, if not essential, element in the development and application of gene drive technology(Target Malaria, 2017; Hartley et al., 2019; Thizy et al., 2019; Kormos et al., 2020; World Health Organization, 2021) The recently-published second edition (2021) of the WHO Guidance for Testing Genetically Modified Mosquitoes, places importance on “a co-development approach that emphasizes authentic partnership and knowledge engagement” for community engagement and development of the technology in general (World Health Organization, 2021). While numerous publications emphasize the importance of co-development, the process of their creation does not regularly apply the practice. We believe that it is essential to include stakeholders and practitioners from field sites in the work of developing guidance and commitments for the development and application of the technology, particularly considering that these are the individuals with whom we should be partnering to ensure a “co-development approach” is applied.

Sharing of knowledge and research and investing in relationships of trust and collaboration are essential in the process of co-developing a shared set of goals and a research pathway to reach those goals. Co-development requires trust (Athaide et al., 2003; Nielsen, 2004; Bidault and Castello, 2019). Trust between research programs and stakeholders and communities. Some recommendations call for a “neutral” third-party facilitator for engagement to avoid potential conflicts of interest, ensure ethically responsible research practice, and maintain balance of power (Kofler et al., 2018). We argue that these recommendations would restrict, if not eliminate, opportunities for true co-development, knowledge sharing and bi-directional communication essential to build trust between a research project and relevant communities. Co-development requires trust and knowledge sharing between these groups; knowledge from communities is essential to inform project practice, principles, target goals, and to establish a balance of power. Recommendations for engagement, and navigation of complex challenges associated with decision-making, and conflicts of interest should be developed at a minimum in consultation with multidisciplinary local experts and those with knowledge and experience in areas where they may be applied.

How do we develop guidelines for best practice and recommendations for ethical engagement and inclusion without partnering with those who are closest to the values and priorities of communities and stakeholder groups that will be affected most by the technology? True belief in co-development calls for co-development of guidelines, which require collaborative work and “authentic partnership” (participants share in the conceptualization, development, and sharing/publication) with a much broader group of stakeholders. This practice begins with an acknowledgement and commitment from the global gene-drive research community to apply co-development practice in the evolution of published guidelines and recommendations, and to place value and trust in the critical perspectives and values of these stakeholder groups. It is essential, for successful co-development of guidelines, to first engage in rigorous knowledge exchange to build capacity of participating groups. This concept of mutual learning and knowledge exchange is well elaborated in the NASEM publication *Genes Drives on the Horizon: Advancing Science, Navigating Uncertainty, and Aligning Research with Public Values* (National Academies of Sciences Engineering and Medicine, 2016). The African Union, New Partnership for Africa’s Development (AUDA-NEPAD) and the Pan-African Mosquito Control Association have initiated important first steps in essential knowledge engagement with potential end-user stakeholders (AUDA-NEPAD, 2018). NEPAD has organized meetings and workshops to discuss gene drives and their potential uses with African stakeholders, and PAMCA (Pan-Africa Mosquito Control Association, 2021), in partnership with Target Malaria (a program developing gene drives for suppressing mosquito populations), has provided training courses on gene drives targeted toward participants that include researchers, policymakers, health professionals, and graduate students. The expansion of knowledge engagement is an essential first step in the facilitation of meaningful co-development of guidelines for potential application.

Without making these efforts, the recommended practices and considerations remain largely reflective of the specific values and ethical concerns of academic experts and institutions, maintaining an imbalance of power and influence.

## Power: Shifting Knowledge-Production and Decision-Making to Local Experts

The subject of power dynamics in gene-drive research has been of particular interest given that the development of the technology is occurring largely in high-income countries (HIC) for deployment in low-to-middle-income countries (LMIC). Large social and economic inequalities between the two, as well as perceptions of historical injustices, are likely to influence the way that knowledge is produced and foreign investment is perceived (National Academies of Sciences Engineering and Medicine, 2016; Rudenko et al., 2018; Kofler and Taitingfong, 2020). Some authors have pointed to inequity and history as potential threats to co-development and the creation of fair and equal partnerships between developers, communities, and governing bodies (Athaide et al., 2003; Nielsen, 20042004; Target Malaria, 2017; Kofler et al., 2018; Rudenko et al., 2018; Bidault and Castello, 2019; Hartley et al., 2019; Kofler and Taitingfong, 2020; Long et al., 2020; Ledingham and Hartley, 2021; Merriam-Webster, 2021). Proposed guidance, frameworks, and webinar discussions offer ways to acknowledge and counterbalance these dynamics and achieve engagement, collaboration, and shared decision making (Matenga et al., 2019; Thizy et al., 2019; Turnhout et al., 2020). As we point out, HIC authors and experts are leading these proposed solutions to inequities in power. Additionally, the specific HIC recommendations for gene-drive research places developers in a position that may require demanding specific criteria or processes that disrespect or disregard local governance and values. It is for these reasons that the inclusion of local perspectives, particularly those of regulators and governing bodies, be integral in the development of proposed guidance/frameworks. An understanding of local governance structures, and how and when they should be involved as partners or as providers of guidance regarding policy and legal frameworks, would assist in the development of guidance that maintains respect for local values. It is important to consider how knowledge is produced and shared, and what information is valued and by whom. A suggested solution to balancing this tension is to position local experts in the lead in knowledge production and decision making (De Graeff et al., 2021). It is legitimate to consider that people aspire to be more than mere spectators in the battles that are being fought in their name, or for their benefit, otherwise it would represent a kind of paternalism or condescension towards them. The best way to achieve authentic partnership with people in any initiative is to involve them in the entire process, under risk of generating skepticism and rejection.

In practice, how do local practitioners and experts take a lead in complex, and competitive practice dominated by the HIC academy? Our proposed first step is their inclusion in the conversations that often lead up to publication of guidance and recommendations. It is up to the current influencers (funders, institutions, and academic experts) in the gene-drive community to put into action the practice of inclusion, co-development, and true engagement that we have articulated as a priority.

## Conclusion

Gene-drive technologies developed for public health applications are complex and present a set of challenging issues that should be explored and considered thoughtfully prior to their application. The uncertainty of outcome of the technology, and the associated risks and benefits, raise important ethical questions and concerns for discussion and exploration. If we are to approach this novel technology in the spirit of true co-development and with determined effort to be inclusive, minimize inequities and imbalances in power, and illuminate the voices and values of those who will be most affected by the application, we need to change our approach to the work. It is essential to include field-site practitioners, stakeholders and community leaders in the academic conversations and debates surrounding these subjects. A new value needs to be placed on reaching these voices and creating a space for sharing their knowledge and prioritizing their perspectives. Without this, the guidelines and recommendations for gene drive presented by the academic community and HIC funders and institutions will fail to meet the ethical goals and commitments they want to achieve.

“Whatever you do for me but without me, you do against me”

Mahatma Gandhi

## Data Availability

The original contributions presented in the study are included in the article/supplementary material, further inquiries can be directed to the corresponding author.
